# The impact of stress on cognitive performance in remote smartphone-based assessments: exploring within-person variability

**DOI:** 10.3389/fdgth.2026.1878732

**Published:** 2026-10-16

**Authors:** Sarah Prieto, Louisa Thompson, Molly Split, Caroline Nester Rooney, Daliah Ross, Preeti Sunderaraman, Alyssa De Vito, Nelson Roque, John M. Felt, Martin J. Sliwinski, Zachary J. Kunicki

**Affiliations:** 1 Department of Psychiatry and Human Behavior, Alpert Medical School of Brown University, Providence, RI, United States; 2 Memory and Aging Program, Butler Hospital, Providence, RI, United States; 3 Department of Psychology, University of South Carolina, Columbia, SC, United States; 4 Department of Psychiatry, Brown University Health, Providence, RI, United States; 5 College of Health and Human Development, The Pennsylvania State University, University Park, PA, United States; 6 Center for Healthy Aging, The Pennsylvania State University, University Park, PA, United States

**Keywords:** app-based, cognitive assessment, contextual factors, digital cognitive assessment, high-frequency assessments, Mobile Monitoring of Cognitive Change, mobile-app, stress

## Abstract

**Introduction:**

Stress is a well-established risk factor for cognitive dysfunction in domains including processing speed (PS), working memory (WM), and episodic memory (EM). However, this research literature typically uses between-group comparisons. Advances in remote, smartphone-based cognitive assessments allow for examination of within-person variability, or testing how fluctuations in an individual's stress levels influence their cognitive performance. We examined the association between within-person variability in stress and performance on mobile-app assessments of processing speed, working memory, and episodic memory in cognitively unimpaired older adults.

**Methods:**

123 cognitively unimpaired older adults (60–81 years old, 66.7% female, 86.2% White, mean education = 16.49 years) rated their stress using a single-item immediately before completing cognitive assessments on the Mobile Monitoring of Cognitive Change (M2C2) smartphone app. Data were gathered three times daily over eight days. Cognitive tasks included measures of PS, visual WM, and EM. Multilevel modeling examined the interaction between within-person stress and session number on cognitive performance.

**Results:**

Higher within-person stress was associated with faster PS (Est. = −3.82, *p* < .001), though these effects diminished across sessions (session × stress interaction, *p* = .003). By contrast, stress was not associated with EM or WM performance. Sensitivity analyses using different cumulative stress thresholds produced consistent findings.

**Discussion:**

Results demonstrate that momentary increases in stress were associated with faster PS, but did not impact WM or EM performances, though these effects changed over repeated testing. These findings highlight the importance of within-person stress variability in shaping cognitive performance on high-frequency smartphone-based assessments.

## 1 Introduction

Increasing evidence suggests that stress is an important correlate of cognitive functioning among older adults. For example, both perceived stress (1–4) and posttraumatic stress disorder (5, 6) are linked to poorer cognitive functioning and accelerated decline over time. Proposed mechanisms underlying the association between stress and cognitive functioning include hypothalamic-pituitary-adrenal (HPA)-axis dysregulation (7), inflammatory pathways (8, 9), and metabolic dysfunction (10), each of which may contribute to poorer cognitive functioning through chronic physiological stress responses. However, evidence from laboratory-based studies and daily-diary research demonstrate that exposure to stress is not uniformly detrimental, and an absence of stress is not consistently associated with better outcomes (11–13). The Yerkes-Dodson framework, for example, suggests that performance actually *increases* until an optimal level of arousal is achieved by increasing attentional resources (14, 15). Although arousal and perceived stress are related but distinct constructs, this framework offers a useful conceptual lens for considering non-linear, dynamic associations between subjective stress and cognitive performance. The present study focuses on subjective, momentary stress (i.e., participants' self-reported perception of their own stress level at the time of assessment) rather than objective stressor exposure or physiological arousal.

Despite the complexity between stress and cognitive aging, the majority of extant work examining associations between these constructs relies on between-person comparisons. Between-person comparisons reflect differences across individuals, such as whether people who report higher average stress levels differ compared to those with lower average stress. In the context of the current study, this would mean comparing individuals who generally report higher stress across the assessment period to those who generally report lower stress. This approach has inherent limitations as it may obscure dynamic ways in which within-person fluctuations in stress influence an individual's cognitive performance in daily life (16). *Within-person effects* capture the differences between an individual's average level of stress and the stress they are feeling at the time of assessment, capturing if someone is feeling more or less stressed than usual (16, 17). In the context of this study, within-person effects examine whether cognitive performance changes during moments when an individual reports feeling more stressed than is typical for them, providing insight into how or moment-to-moment fluctuations in stress relate to cognitive functioning.

Because within-person effects are inherently dynamic, understanding their influence on cognition requires repeated assessments conducted close in time to when they occur. Advances in high-frequency digital-based testing provide a methodological solution to this challenge (18, 19). Unlike traditional neuropsychological evaluations that provide a snapshot of performance in time, digital tools allow for the implementation of brief cognitive assessments that can capture performance across and within several days. A small but growing body of research has linked momentary stress to cognitive performance. For example, elevated daily stress has been associated with slower reaction time on demanding working-memory tasks (20), while a greater number of daily stressors has been linked to poorer perceived cognitive performance via ecological momentary assessment (21). The present study builds on this work by pairing high-frequency stress ratings with objective, standardized cognitive tasks delivered via a validated smartphone-based platform.

Performance during traditional neuropsychological evaluations may also be impacted by factors including unfamiliar environments or fatigue from lengthy testing sessions, whereas brief at-home digital cognitive assessments may better reflect typical functioning. These high-frequency assessments (HFA) enable tracking of subtle changes that may otherwise go undetected (22). Because digital tools are highly scalable and cost-effective, they are particularly promising for early detection of cognitive impairment, as well as longitudinal monitoring (23). Using HFA provides a unique opportunity to examine within-person deviations and how real-world contextual factors, such as stress, dynamically shape cognitive performance, offering a more nuanced understanding of cognition in daily life (24). Repeated assessments not only facilitate examination of within-person stress-cognition associations but also provide insight into intraindividual variability in cognitive performance. Specifically, examining within-person variability using HFA may reveal early indicators of cognitive risk that would otherwise be obscured when relying solely on between-person effects. For example, prior work showed that individuals with mild cognitive impairment exhibit greater intraindividual variability than cognitively unimpaired older adults on tasks of processing speed and visual short-term memory binding (25). In that study, Cerino et al. suggested that this variability may be a meaningful marker of cognitive vulnerability, but noted that they did not examine psychosocial factors, such as perceived stress, that may actually fluctuate from session to session. They called for future research examining how these time-varying factors relate to variability in HFA performance. At the same time, variability in performance can be shaped by environmental and contextual factors (26). HFA are particularly well-suited for studying stress, as they can capture the immediate cognitive consequences of momentary stress that retrospective stress measures may overlook (27, 28). Understanding state-related influences may aid in accurate interpretation of cognitive data. In other words, it is important to measure contextual influences, such as elevations in perceived stress, because they may affect cognitive variability and, in turn, how session-to-session variability is interpreted as a marker of risk or vulnerability. Incorporating information about contextual factors may enhance the sensitivity and validity of digital cognitive assessments among older adults (29).

Despite advances in digital testing, relatively little is known about how momentary fluctuations in stress influence different cognitive domains during HFA in cognitively unimpaired older adults. In the current study, we investigated associations between fluctuations in stress and within-person cognitive performance on tasks of processing speed, working memory, and episodic memory using HFA. We hypothesized that higher self-reported stress would generally predict poorer cognitive performance. We also examined which cognitive domains (processing speed, working memory, and episodic memory) were most sensitive to stress fluctuations. These findings may inform the interpretation of digital cognitive assessments in real-world contexts and guide future validation efforts of mobile cognitive tools. Moreover, this approach has potential as a method for monitoring dynamic cognitive functioning across the lifespan.

## 2 Method

### 2.1 Participants and recruitment

Participants were recruited from the Butler Hospital Memory and Aging Program Alzheimer's Prevention Registry, which is a database of older adults living in the northeastern United States interested in participating in dementia-related research (30). Participants were eligible if they were 60 years or older and had familiarity with the basic features of smartphones. Exclusion criteria included self-reported history of cognitive impairment or dementia, history of neurologic disease or severe mental illness, and physical inability to complete smartphone-based testing. A total of 256 individuals were contacted via email and/or telephone. Of these, 146 interested individuals engaged in the Modified Telephone Interview for Cognitive Status (TICS-M) (31). The remaining individuals did not respond or declined to participate. The TICS-M ranges from 0 to 50, with lower scores indicating worse cognitive functioning. To be eligible, participants had to demonstrate unimpaired cognition, defined using a previously established cutoff score of ≥34 (31). Although the TICS-M has demonstrated strong performance for identifying broad cognitive impairment, its ability to differentiate MCI from normal cognition is limited, and alternative thresholds have been proposed (32). Because the goal of the present study was to enroll cognitively unimpaired adults rather than classify MCI, the ≥34 threshold was considered appropriate. During screening, 23 participants were excluded, and 1 participant was lost to follow up following the screening. *N* = 123 cognitively unimpaired adults between the ages of 60 and 81 years old ultimately enrolled in the study. Additional recruitment information is available in published work (33, 34).

### 2.2 Ethical considerations

The project was approved by the Butler Hospital Institutional Review Board (#1882523). Participants provided informed consent, and a $20 gift card was provided as compensation for their time. To safeguard participant information, data were de-identified.

### 2.3 Mobile-app based cognitive assessment

#### 2.3.1 Overview

Android smartphones (Motorola Moto G8 Play; Android version 10 through 13) preloaded with the Mobile Monitoring of Cognitive Change (M2C2) app were shipped to participants along with a detailed user guide (35). Remote cognitive tasks were completed three times daily over eight days, yielding up to 24 assessments per participant. The brief cognitive battery included tests of processing speed (Symbol Match), working memory (Color Shapes), and episodic memory (Prices) developed for M2C2 and described previously; See Supplementary Figure S1 and Hakun et al. for details (34, 35). Each task took approximately 60 s to complete, consistent with prior HFA study protocols using the M2C2 app (19, 25).

#### 2.3.2 Cognitive domains

Processing speed was assessed using the Symbol Match task, an established timed continuous performance measure consisting of a two-alternative forced choice paradigm in which participants identify matching symbol pairs across 16 trials (19, 36, 37). Performance was measured by the median reaction time across correct trials in milliseconds, as well as the proportion of correct responses.

Working memory was assessed with the Color Shapes task, a visual change detection task measuring intra-item feature binding (38, 39). Participants determine whether shapes changed color across two sequential presentations, with shape locations also changing. Performance was measured using discriminability (d'), calculated from the proportion of correct identifications and the false alarm rate (40).

Episodic memory was assessed via the Prices task. In this task, participants incidentally encoded ten grocery item–price pairs while judging whether each price was “good” (41, 42). This was followed by delayed forced-choice recognition trials in which participants were asked to identify the previously seen price associated with each grocery item. Performance was measured by the proportion of correct responses on recognition trials.

Cognitive performance was examined for outliers using ±3 SD thresholds applied at the sample level (i.e., values falling more than 3 SDs above or below the overall sample mean for each variable were flagged). Accuracy, proportion correct, and d′ values were retained given that all fell within plausible performance ranges. In contrast, 29 extreme reaction time values on the Symbol Match task (>3 SD above the mean) were recoded to the upper 3 SD cutoff. A 3 SD cutoff was selected because prior simulation work indicates that standard deviation–based approaches reduce the influence of extreme reaction times while introducing relatively little bias compared with alternative approaches (43). Winsorization was preferred over trial exclusion because it preserves the total number of observations available and avoids introducing missingness that could bias estimates. Recoding these values to the 3 SD upper boundary retains their directional information while limiting undue influence on parameter estimates. Descriptive statistics for cognitive performance are presented in Supplementary Table S1.

### 2.4 Stress assessments

Participants rated their stress levels immediately before completing cognitive testing using the in-app prompt: “*Please rate your current stress level*.” Stress ratings ranged from 0 (“not at all stressed”) to 100 (“extremely stressed”) and were assessed immediately before each testing session. To quantify perceived stress elevations, we first calculated a mean stress score separately for each participant across all observations. Momentary stress ratings were compared to this individual mean. Because no consensus threshold exists for defining elevated momentary stress in HFA studies, stress was operationalized as occurring when a participant's rating exceeded 0.5 standard deviations above each person's individual mean. We selected 0.5 SD as a threshold intended to be sensitive enough to capture a sufficient number of within-person stress deviations per participant, while minimizing assumptions about an individual's absolute stress level. Similar within-person deviation approaches using a 0.5 SD threshold have been used to identify departures from typical affective states (44). We generated a time-varying cumulative stress tally for each participant reflecting the number of instances when their reported stress surpassed this threshold. This approach operationalizes stress as within-person deviations (i.e., time-varying differences from an individual's own mean level), rather than as a summary measure of within-person variability. Sensitivity analyses examined alternative thresholds of 1.0 and 1.5 SD to evaluate whether findings were robust to increasingly conservative definitions of elevated within-person stress.

### 2.5 Statistical analysis

Descriptive statistics were used to characterize the sample in terms of demographics, objective cognitive performance, and stress. Linear mixed-effects models, using the ***nlme*** package in R version 4.3.1 (45), were used to assess the relations between stress and cognition. To account for the possibility that observations collected closer in time are expected to be more highly correlated than those further apart (i.e., temporal autocorrelation), we specified an autoregressive correlation structure within participants (46). Covariates included session number, time of day, age, education, and sex. Session number refers to the sequential number of each cognitive testing session (1–24) across the eight study days, while time of day includes morning, afternoon, or evening. Age and education were centered prior to model estimation to aid in interpretation. In addition, a binary indicator of first session (vs. all subsequent sessions) was included as a covariate to account for potential practice effects (36). Models included random intercepts, to account for between-person differences in baseline performance, and random slopes for cumulative stress exposure, to allow individual differences in stress accumulation over time. The ***effectsize*** package in R was used to obtain standardized effects.

## 3 Results

### 3.1 Participant characteristics

See Table 1 for demographic and sample characteristics. The sample included 123 individuals, with a mean age of 68.86 years (SD = 4.94; range = 60–81 years). Our sample was primarily White (86.2%; *n* = 106) and female (66.7%; *n* = 82). As noted above, participants were able to engage in a maximum of 24 sessions (i.e., 3 sessions per day for 8 days). Adherence was high in our sample; participants completed an average of 95.6% of sessions (SD = 12.67). Participants rated an average stress level of 27.43 (SD = 23.93, range = 0–100) across sessions.

**Table 1:** Sample characteristics.

Characteristics	*n* (%), M (SD)	Range
Sex, *n* (%)		
Male	41 (33.3)	
Female	82 (66.7)	
Age (years), M (SD)	68.9 (4.9)	60–81
Education—M, (SD)	16.5 (2.4)	12–21
Missing	2 (1.6)	
Race, *n* (%)		
American Indian or Alaska Native	1 (0.81)	
Asian	1 (0.81)	
Black or African American	4 (3.25)	
White	106 (86.18)	
More than one race	6 (4.88)	
Other	1 (0.81)	
Unknown or not reported	4 (3.25)	

M, mean; SD, standard deviation.

### 3.2 Association between stress and processing speed

Results from the linear mixed effects model predicting symbol search performance reaction time, operationalized as median response time on correct trials, are presented in Tables 2, 3 and Figure 1. Variance inflation factors (VIFs) ranged from 1.02 (between-person stress) to 4.09, with the highest values observed for within-person stress and the session × within-person stress interaction, indicating that multicollinearity among predictors was not a concern. VIF values of 5 or higher are commonly used as an indicator of potentially problematic multicollinearity and may need to be omitted from the models (47). Therefore, all predictors were retained in the final models.

**Table 2:** Linear mixed effects model results for processing speed (in ms).

Variable	Reference group	Est.	Lower 95% CI	Upper 95% CI	*p*-value
Between-Person Stress	—	0.59	−3.46	4.64	.78
Session Number	—	−28.33	−32.57	−24.10	<.001
Within-Person Stress	—	−3.74	−5.51	−1.98	<.001
Education (centered)	—	−25.99	−57.89	5.91	.11
Sex (female vs. male)	Male	13.02	−147.41	173.44	.87
Age (centered)	—	41.19	25.74	56.65	<.001
Time of day (evening vs. afternoon)	Afternoon	82.50	53.91	111.09	<.001
Time of day (morning vs. afternoon)	Afternoon	18.72	−10.24	47.69	.21
Session (Subsequent vs. First)	First session	−427.81	−494.25	−361.36	<.001
Cumulative Stress (0.5 SD)	—	4.19	−14.97	23.35	.67
Session × Within-Person Stress	—	0.23	0.09	0.36	.001

Unstandardized effects (Est.) reported. Random Effect for Intercept, *σ* = 466.93; Random slope, *σ* = 47.62.

**Table 3:** Summary of standardized effects for processing speed reaction time.

Stress effect (SD)	Standardized effect (95% CI)
Between-person effect	
0.5 SD	0.02 (−0.11, 0.14)
1.0 SD	0.03 (−0.09, 0.16)
1.5 SD	0.03 (−0.10, 0.16)
Within person effect	
0.5 SD	−0.02 (−0.05, −0.002)
1.0 SD	−0.02 (−0.04, 0.0009)
1.5 SD	−0.03 (−0.05, −0.004)
Session × Within-Person Stress	
0.5 SD	0.04 (0.02, 0.06)
1.0 SD	0.04 (0.02, 0.06)
1.5 SD	0.03 (0.01, 0.06)

Standardized effects are calculated by refitting each model using standardized variables, so these results are similar to standardized regression coefficients where 0.10, 0.30, and 0.50 are considered small, medium, and large effect sizes, respectively.

**Figure 1:**
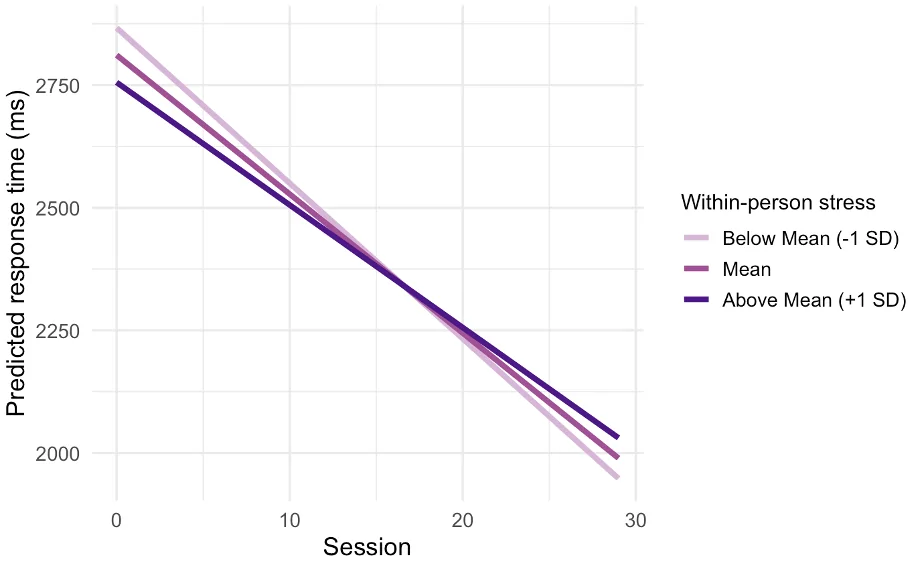
Interaction between time in study and processing speed performance. The figure displays predicted processing speed (ms; *y*-axis) across session number (*x*-axis). Dark purple = 0.5 SD above the mean; medium purple = at the mean; light purple = 0.5 SD below the mean. Predictions are based on the linear mixed model, with all other covariates held at their average or reference values. Higher within-person stress predicted faster processing speed, but this effect weakened over time.

Consistent with our hypothesis, a significant interaction between session and within-person stress emerged [Estimate (Est.) = 0.23, *p* = .001], indicating that the positive association between within-person stress and response time diminished across testing sessions. Significant main effects were observed for session (Est. = −28.33, *p* < .001), within-person stress (Est. = −3.74, *p* < .001), and age (Est. = 41.19, *p* < .001). These effects and additional covariate estimates are presented in Table 2. Findings suggest that participants generally responded more quickly as the study progressed, during periods of elevated stress relative to their own average, and more slowly with increasing age. Johnson–Neyman analyses indicated the association between elevated within-person stress and faster responding was strongest earlier in the study. Specifically, higher-than-usual within-person stress was significantly associated with reaction time during earlier sessions (<12.6). The results were no longer statistically significant from 12.6 sessions to the end of the follow-up period. In our sensitivity analyses using alternative thresholds for cumulative stress events (1 and 1.5 SD), we consistently saw a positive association between stress events and processing speed, but no result achieved statistical significance. The interaction effect remained relatively stable in each model, and each effect was very small as they did not reach the threshold for a small effect (i.e., 0.10) in Cohen's taxonomy. See Supplementary Tables S2, S3.

We also examined symbol search accuracy, calculated as the proportion of correct trials out of the total number of trials. Results from the linear mixed effects model predicting accuracy are shown in Supplementary Figure S2 and Tables S4–S6. A significant interaction between session and within-person stress was observed at all thresholds (Est. = 0.0019, *p* = .035), indicating that the negative association between stress and accuracy lessened slightly across sessions. Significant main effects were found for within-person stress (Est. = −0.025, *p* = .042) and session (first vs. subsequent; Est. = −1.34, *p* = .005), indicating that accuracy was lower during participants’ first session than during all subsequent sessions. Sensitivity analyses using cumulative stress thresholds of 1 and 1.5 SD yielded similar patterns, with the session × within-person stress interaction remaining consistent. These results suggest a complementary pattern between reaction time and accuracy in the symbol search task. While participants generally responded faster as sessions progressed, accuracy increased across sessions among participants experiencing higher within-person stress.

### 3.3 Association between stress and working memory

Supplementary Table S7 details results from the linear mixed effects model examining predictors of working memory. Neither within-person nor between-person stress significantly predicted working memory, and the session × within-person stress interaction was not significant. Sensitivity analyses at different cumulative stress thresholds yielded similar null findings (Supplementary Tables S8, S9).

### 3.4 Association between stress and episodic memory

Supplementary Table S10 summarizes results from the linear mixed effects model examining predictors of performance on episodic memory (Prices task). Stress variables (i.e., within-person, between-person, and cumulative stress) were not significantly related to episodic memory, nor was the session × within-person stress interaction significant. Sensitivity analyses provided consistent results (Supplementary Tables S11, S12).

## 4 Discussion

We sought to examine within-person associations between stress and cognitive performance using remote, smartphone-based HFA. By leveraging repeated measures across eight days, we examined how fluctuations in stress influence performance on tasks of processing speed, working memory, and episodic memory. Results suggest that stress is differentially associated with performance across cognitive domains, such that within-person increases in stress were associated with faster reaction time and reduced accuracy within a processing speed task. Importantly, this association shifted across sessions such that higher momentary stress was associated with faster processing speed early in the study, but slower processing speed by the final session. This indicates that the impact of momentary stress is dynamic and may attenuate with repeated exposures. By contrast, stress fluctuations were not associated with working memory or episodic memory. These findings highlight the importance of considering within-person deviations rather than relying solely on between-person comparisons to capture the dynamic ways that stress shapes cognition.

Prior research investigating the effects of stress on cognition has largely relied on between-group differences, often comparing individuals with high-vs.-low stress. Although such studies provide valuable insights, they do not account for unique within-person deviations that reflect natural fluctuations in stress levels. Among the relatively few studies that have examined within-person variability, results tend to show more consistent effects of stress on processing speed relative to other cognitive domains, including working memory (20, 48). This heightened vulnerability may be because processing speed performance depends on fast and efficient recruitment of attentional resources, which may be negatively impacted by the body's response to stress and activates the sympathetic nervous system and the HPA axis (7).

Our findings add to the existing literature by demonstrating that relatively low stress levels were associated with faster processing speed performance over time. While this appears to contradict prior findings, which show reductions in processing speed in the setting of high stress levels, we hypothesize that these findings in tandem relate to the hypothesis of a dynamic association between stress and processing speed (14, 15). This hypothesis suggests that the right amount of stress can improve performance, while high stress or even too little stress can dampen it. Our findings may reflect a context in which mild stress in a clinically unimpaired sample is associated with a more “optimal level” of stress, wherein higher stress leads to faster processing speed. In addition to considering speed, these reaction time findings should be considered alongside our accuracy results. Within-person increases in stress were associated with a small but statistically significant reduction in accuracy on the same processing speed task, and this association attenuated across sessions. In other words, early in the study, momentary increases in stress were associated with faster but slightly less accurate responding, but over time, both associations weakened and trended in the opposite direction. Together, these patterns are consistent with a speed-accuracy trade-off. Another plausible hypothesis of the observed association that warrants further investigation is that stress may facilitate performance on more novel or challenging tasks, whereas familiar tasks may be less influenced. The observed attenuation across sessions may also suggest that practice effects, particularly in speeded tasks, progressively overshadow transient arousal benefits as participants become more efficient with task demands. Because practice effects are a well-documented phenomenon that may influence performance across sessions (49–51), our analyses adjusted for session number and a binary indicator distinguishing the first assessment from all later assessments.

Working memory performance, by contrast, was not associated with within-person stress fluctuations in the present study. This may reflect the observation that stress-related impairments in working memory are most often reported under conditions of acute, high-intensity stress, likely because of the prefrontal cortex's sensitivity to elevated stress responses (42). As such, the naturalistic short-term fluctuations in stress assessed here may have been insufficient to adversely affect working memory.

Similar to working memory, episodic memory performance also remained stable. One possible explanation is that disruptions in episodic memory may only occur after exposure to chronic or highly stressful events rather than brief, momentary spikes (52). This is because sustained elevations of cortisol tend to arise after exposure to prolonged stress which has been associated with poorer episodic memory over time, mediated by neurotoxic hippocampal injury (53). Given that the hippocampus has a high metabolic demand relative to other brain structures, prolonged stress can lead to oxidative damage and disrupt neuronal connectivity, resulting in impaired memory formation (54–57). Because stress levels in our sample were assessed over short intervals and in response to normal daily life, it is plausible that stress did not reach the threshold necessary to disrupt memory processes. It is also possible that stress-induced memory impairment depends on the timing of the stress exposure relative to the phase of memory processing. For example, stress immediately before or during encoding may enhance (or in some cases diminish) memory formation depending on the emotional salience of the material, whereas stress during consolidation or retrieval is more likely to impair performance. As such, the lack of association between episodic memory and stress fluctuations may reflect both the timing of stress exposure and task-dependent differences in stress effects on episodic memory.

These findings have several implications for the use of digital cognitive tools. First, they underscore the importance of monitoring contextual factors, such as stressful experiences, when interpreting digital cognitive performance. Extant work using EMA has shown the value of using real-time assessments of depressive symptoms and sleep quality to understand cognitive functioning (58, 59). Consistent with work encouraging neuropsychologists to leverage tools that enable repeated assessments and account for contextual factors to improve the sensitivity and ecological validity of cognitive measurement (60), our results demonstrate that momentary stress fluctuations can meaningfully impact performance. Given that stress can influence processing speed, failing to monitor and methodically account for momentary fluctuations may lead to misinterpretation of cognitive data. When conducting research or clinical work with older adult populations to facilitate early detection of cognitive decline, it is important to consider the possibility that even modest fluctuations in stress may influence cognitive performance, potentially masking or exacerbating deficits, which can complicate diagnostic conclusions. Incorporating real-time stress assessments via smart-phones, or objective measures such as passive sensing or physiological metrics, could yield more precise measurement of stress and enhance the accuracy and ecological validity of digital monitoring tools. Accounting for stress in real-world data collection could improve the sensitivity of early cognitive screening, offer a more accurate detection of subtle cognitive difficulties, and potentially enhance methods for identifying individuals at risk for dementia.

### 4.1 Limitations

Several limitations warrant consideration. Stress was assessed with a single-item self-report measure, which may not fully capture its complexity (e.g., momentary vs. chronic stress, fluctuations across days, or cumulative stress exposure over time). More frequent assessments would allow researchers to clarify when and for whom stress most strongly impacts cognition. In particular, examining lagged effects (i.e., how stress at one timepoint influences cognitive performance at a later session) would be informative. In addition, our operationalization of stress as the accumulation of within-person deviations above a person's mean is not sensitive to consistently elevated stress levels or high stress variability and may therefore underestimate overall stress burden in some individuals.

Regarding other methodological considerations, reaction times were winsorized rather than excluded. Though winsorization is a commonly used approach in psychological research for handling extreme values while preserving sample size, it may be less appropriate if extreme values reflect invalid responses. In this analysis, we operated under the assumption that all extreme values were valid responses and were not due to some other issue (e.g., the participant put their phone down after beginning the task). However, sensitivity analyses repeating the primary reaction time analyses after excluding extreme reaction time values yielded similar results: the within-person stress × session interaction remained significant (*β* = 0.16, SE = 0.07, *p* = .015), indicating that the observed association was not driven by extreme reaction time observations. Future research may need to account for ways to determine if participants were interrupted before completing the task to determine valid vs. invalid responses. Another limitation includes the modeling of practice effects using a binary first-vs.-subsequent session indicator. While practical, this approach does not fully capture the nuanced nature of practice effects, and future work using other approaches may better characterize learning dynamics in high-frequency assessments. Additionally, participants used Android devices, all of the same model, running the most up-to-date version of the Android OS, and we did not formally account for OS version as a covariate. Prior work suggests device and OS variability can affect timing precision in mobile assessments (61), and future studies should consider documenting and controlling for this.

Other contextual influences, such as sleep quality, mood, or environmental distractions, were not systematically measured but may play an important role in shaping daily cognitive performance (62, 63). We similarly did not assess family structure (e.g., marital/partner status, living arrangement), which prior research suggests can influence stress exposure and reactivity (64–66). Future studies should consider this as an additional contextual factor, and they can work towards examining the diversity of emotional experiences on cognitive performance (67). Moreover, our operationalization of elevated stress using thresholds based on deviations from each individual's mean was not pre-specified due to an *a priori* rationale. Though sensitivity analyses suggest robustness of results across alternative thresholds, this approach may not fully capture cumulative or consistently high stress. Another limitation is that our sample was primarily White and highly educated, which may limit generalizability to more heterogeneous populations. Finally, our analyses focused on cognitively unimpaired older adults; future research which extends this work to individuals experiencing mild cognitive impairment will be critical for evaluating the utility of within-person stress deviations as an early marker of disease.

## 5 Conclusions

Digital tools offer a potentially more accurate and ecologically valid method for assessing cognition in older adults and are growing in popularity among early detection research. Our findings underscore that it is essential to consider the impact of contextual factors, including stress, on cognitive performance, particularly when assessing processing speed, to ensure diagnostic conclusions are adequately informed when using these tools. Moreover, evaluating cognitive performance using within-person comparisons provides greater insight into how fluctuations in stress and other contextual factors (i.e., mood) may influence cognitive variability over time at the individual level. Future research is encouraged to integrate real-time stress assessments with longitudinal cognitive monitoring to provide greater understanding of whether this approach can identify individuals at risk for cognitive decline that may otherwise go undetected when using traditional cognitive assessment. By better accounting for intra-individual variability, researchers and clinicians may capture early signals of cognitive decline more readily, supporting the capacity to provide more timely intervention.

## Data Availability

The raw data supporting the conclusions of this article will be made available by the authors, without undue reservation.
