# Supplementary material for: The impact of stress on cognitive performance in remote smartphone-based assessments: exploring within-person variability
Source: Front Digit Health. 2026 Oct 16;8:1878732. doi: 10.3389/fdgth.2026.1878732 (PMC13720768; doi:10.3389/fdgth.2026.1878732)
Supplement: Supplementary file 1 [file Table1.docx]

**Supplementary Tables and Figures**

Table S1. Descriptive Statistics for Cognitive Performance Variables

| **Variable** | **M** | **SD** | **Min** | **Max** |
| --- | --- | --- | --- | --- |
| Symbol search performance reaction time | 2296.47 | 654.44 | 1057.00 | 6912.50 |
| Symbol search accuracy | 96.57 | 4.96 | 62.50 | 100.00 |
| Color Shapes d' | 2.76 | 1.19 | −2.07 | 4.65 |
| Prices Proportion Correct | 0.75 | 0.16 | 0.00 | 1.00 |

Note: M = mean; SD = standard deviation; RT = reaction time; d′ = discriminability. Values reflect session-level observations prior to winsorization of extreme reaction times.

Table S2. Linear Mixed Effects Model Results for Processing Speed at 1 SD

| **Variable** | **Reference Group** | **Est.** | **Lower**  **95% CI** | **Upper**  **95% CI** | **p-value** |
| --- | --- | --- | --- | --- | --- |
| **Between-Person Stress** | - | 1.11 | -2.96 | 5.19 | .59 |
| **Session Number** | - | -25.92 | -29.53 | -22.31 | <.001 |
| **Within-Person Stress** | - | -3.58 | -5.35 | -1.80 | <.001 |
| **Education (centered)** | - | -23.20 | -55.25 | 8.86 | .16 |
| **Sex (female vs. male)** | Male | -5.51 | -166.61 | 155.58 | .95 |
| **Age (centered)** | - | 40.21 | 24.71 | 55.71 | <.001 |
| **Time of day (evening vs. afternoon)** | Afternoon | 82.35 | 53.77 | 110.92 | <.001 |
| **Time of day (morning vs. afternoon)** | Afternoon | 19.31 | -9.65 | 48.26 | .19 |
| **Session (Subsequent vs. First)** | First session | -422.86 | -489.71 | -356.01 | <.001 |
| **Cumulative Stress (1.0 SD)** | - | -10.78 | -38.64 | 17.08 | .45 |
| **Session × Within-Person Stress** | - | 0.22 | 0.09 | 0.36 | .001 |

Random Effect for Intercept, σ 450.26; Random slope, σ = 74.13. Note: Unstandardized effects (Est.) reported.

Table S3. Linear Mixed Effects Model Results for Processing Speed at 1.5 SD

| **Variable** | **Reference Group** | **Est.** | **Lower**  **95% CI** | **Upper**  **95% CI** | **p-value** |
| --- | --- | --- | --- | --- | --- |
| **Between-Person Stress** | - | 0.97 | -3.27 | 5.21 | .65 |
| **Session Number** | - | -26.66 | -29.75 | -23.58 | <.001 |
| **Within-Person Stress** | - | -3.53 | -5.31 | -1.75 | <.001 |
| **Education (centered)** | - | -25.77 | -58.62 | 7.08 | .12 |
| **Sex (female vs. male)** | Male | -17.11 | -181.41 | 147.20 | .84 |
| **Age (centered)** | - | 39.56 | 23.72 | 55.40 | <.001 |
| **Time of day (evening vs. afternoon)** | Afternoon | 81.55 | 53.04 | 110.05 | <.001 |
| **Time of day (morning vs. afternoon)** | Afternoon | 19.25 | -9.64 | 48.14 | .19 |
| **Session (Subsequent vs. First)** | First session | -416.75 | -484.07 | -349.42 | <.001 |
| **Cumulative Stress (1.5 SD)** | - | -6.15 | -39.91 | 27.60 | .72 |
| **Session × Within-Person Stress** | - | 0.20 | 0.07 | 0.34 | .003 |

Random Effect for Intercept, σ = 432.281; Random slope, σ = 80.675. Note: Unstandardized effects (Est.) reported.

Table S4. Linear Mixed Effects Model Results for Symbol Search Accuracy at 0.5 SD

| **Variable** | **Reference Group** | **Est.** | **Lower**  **95% CI** | **Upper**  **95% CI** | **p-value** |
| --- | --- | --- | --- | --- | --- |
| **Between-Person Stress** | - | -0.01 | -0.04 | 0.01 | .30 |
| **Session Number** | - | 0.01 | -0.04 | 0.06 | .80 |
| **Within-Person Stress** | - | -0.03 | -0.05 | -0.001 | .04 |
| **Education (centered)** | - | 0.02 | -0.16 | 0.21 | .80 |
| **Sex (female vs. male)** | Male | 0.32 | -0.58 | 1.23 | .50 |
| **Age (centered)** | - | -0.04 | -0.13 | 0.05 | .40 |
| **Time of day (evening vs. afternoon)** | Afternoon | 0.13 | -0.29 | 0.55 | .60 |
| **Time of day (morning vs. afternoon)** | Afternoon | -0.11 | -0.54 | 0.31 | .60 |
| **Session (Subsequent vs. First)** | First session | -1.34 | -2.26 | -0.41 | .01 |
| **Cumulative Stress (0.5 SD)** | - | -0.01 | -0.19 | 0.16 | .90 |
| **Session × Within-Person Stress** | - | 0.002 | 0.0001 | 0.004 | .04 |

Note: Unstandardized effects (Est.) reported. Random Effect for Intercept, σ = 2.07; Random slope, σ = 0.18

Table S5. Linear Mixed Effects Model Results for Symbol Search Accuracy at 1 SD

| **Variable** | **Reference Group** | **Est.** | **Lower**  **95% CI** | **Upper**  **95% CI** | **p-value** |
| --- | --- | --- | --- | --- | --- |
| **Between-Person Stress** | - | -0.01 | -0.03 | 0.01 | .34 |
| **Session Number** | - | -0.02 | -0.06 | 0.02 | .34 |
| **Within-Person Stress** | - | -0.03 | -0.05 | -0.002 | .03 |
| **Education (centered)** | - | 0.03 | -0.13 | 0.19 | .70 |
| **Sex (female vs. male)** | Male | 0.44 | -0.37 | 1.25 | .29 |
| **Age (centered)** | - | -0.04 | -0.12 | 0.04 | .34 |
| **Time of day (evening vs. afternoon)** | Afternoon | 0.13 | -0.29 | 0.55 | .54 |
| **Time of day (morning vs. afternoon)** | Afternoon | -0.12 | -0.55 | 0.30 | .57 |
| **Session (Subsequent vs. First)** | First session | -1.36 | -2.28 | -0.43 | .004 |
| **Cumulative Stress (1.0 SD)** | - | 0.21 | -0.05 | 0.46 | .11 |
| **Session × Within-Person Stress** | - | 0.002 | 0.0002 | 0.004 | .03 |

Random Effect for Intercept, σ = 2.68; Random slope, σ = 0.55. Note: Unstandardized effects (Est.) reported.

Table S6. Linear Mixed Effects Model Results for Symbol Search Accuracy at 1.5 SD

| **Variable** | **Reference Group** | **Est.** | **Lower**  **95% CI** | **Upper**  **95% CI** | **p-value** |
| --- | --- | --- | --- | --- | --- |
| **Between-Person Stress** | - | -0.01 | -0.03 | 0.01 | .37 |
| **Session Number** | - | -0.01 | -0.05 | 0.02 | .55 |
| **Within-Person Stress** | - | -0.03 | -0.05 | -0.002 | .04 |
| **Education (centered)** | - | 0.02 | -0.15 | 0.19 | .79 |
| **Sex (female vs. male)** | Male | 0.49 | -0.34 | 1.32 | .25 |
| **Age (centered)** | - | -0.02 | -0.11 | 0.06 | .57 |
| **Time of day (evening vs. afternoon)** | Afternoon | 0.13 | -0.29 | 0.55 | .54 |
| **Time of day (morning vs. afternoon)** | Afternoon | -0.11 | -0.54 | 0.31 | .60 |
| **Session (Subsequent vs. First)** | First session | -1.34 | -2.27 | -0.41 | .01 |
| **Cumulative Stress (1.5 SD)** | - | 0.21 | -0.05 | 0.47 | .12 |
| **Session × Within-Person Stress** | - | 0.002 | 0.0001 | 0.004 | .04 |

Random Effect for Intercept, σ = 2.38; Random slope, σ = 0.36.

Note: Unstandardized effects (Est.) reported.

Table S7. Linear Mixed Effects Model Results for Working Memory

| **Variable** | **Reference Group** | **Est.** | **Lower**  **95% CI** | **Upper**  **95% CI** | **p-value** |
| --- | --- | --- | --- | --- | --- |
| **Between-Person Stress** | - | -0.0002 | -0.01 | 0.01 | .96 |
| **Session Number** | - | 0.04 | 0.03 | 0.05 | <.001 |
| **Within-Person Stress** | - | -0.002 | -0.01 | 0.002 | .33 |
| **Education (centered)** | - | 0.01 | -0.05 | 0.07 | .74 |
| **Sex (female vs. male)** | Male | 0.35 | 0.07 | 0.64 | .02 |
| **Age (centered)** | - | -0.05 | -0.07 | -0.02 | .001 |
| **Time of day (evening vs. afternoon)** | Afternoon | 0.04 | -0.04 | 0.12 | .30 |
| **Time of day (morning vs. afternoon)** | Afternoon | 0.10 | 0.02 | 0.18 | .02 |
| **Session (Subsequent vs. First)** | First session | 0.50 | 0.32 | 0.68 | <.001 |
| **Cumulative Stress (0.5 SD)** | - | -0.01 | -0.05 | 0.03 | .77 |
| **Session × Within-Person Stress** | - | 0.0002 | -0.0002 | 0.001 | .27 |

Random Effect for Intercept, σ = 0.72; Random slope, σ = 0.075. Note: Unstandardized effects (Est.) reported.

Table S8. Linear Mixed Effects Model Results for Working Memory at 1 SD

| **Variable** | **Reference Group** | **Est.** | **Lower**  **95% CI** | **Upper**  **95% CI** | **p-value** |
| --- | --- | --- | --- | --- | --- |
| **Between-Person Stress** | - | -0.001 | -0.01 | 0.01 | .81 |
| **Session Number** | - | 0.04 | 0.03 | 0.04 | <.001 |
| **Within-Person Stress** | - | -0.002 | -0.01 | 0.002 | .31 |
| **Education (centered)** | - | 0.01 | -0.04 | 0.07 | .65 |
| **Sex (female vs. male)** | Male | 0.38 | 0.09 | 0.67 | .01 |
| **Age (centered)** | - | -0.05 | -0.07 | -0.018 | .002 |
| **Time of day (evening vs. afternoon)** | Afternoon | 0.04 | -0.04 | 0.12 | .31 |
| **Time of day (morning vs. afternoon)** | Afternoon | 0.10 | 0.02 | 0.18 | .02 |
| **Session (Subsequent vs. First)** | First session | 0.50 | 0.32 | 0.68 | <.001 |
| **Cumulative Stress (1.0 SD)** | - | -0.01 | -0.07 | 0.05 | .80 |
| **Session × Within-Person Stress** | - | 0.0002 | -0.0002 | 0.001 | .25 |

Random Effect for Intercept, σ = 0.75; Random slope, σ = 0.11. Note: Unstandardized effects (Est.) reported.

Table S9. Linear Mixed Effects Model Results for Working Memory at 1.5 SD

| **Variable** | **Reference Group** | **Est.** | **Lower**  **95% CI** | **Upper**  **95% CI** | **p-value** |
| --- | --- | --- | --- | --- | --- |
| **Between-Person Stress** | - | -0.001 | -0.01 | 0.01 | .71 |
| **Session Number** | - | 0.04 | 0.03 | 0.04 | <.001 |
| **Within-Person Stress** | - | -0.003 | -0.007 | 0.002 | .28 |
| **Education (centered)** | - | 0.02 | -0.04 | 0.08 | .50 |
| **Sex (female vs. male)** | Male | 0.39 | 0.11 | 0.68 | .01 |
| **Age (centered)** | - | -0.05 | -0.07 | -0.02 | .001 |
| **Time of day (evening vs. afternoon)** | Afternoon | 0.04 | -0.04 | 0.12 | .29 |
| **Time of day (morning vs. afternoon)** | Afternoon | 0.10 | 0.03 | 0.18 | .02 |
| **Session (Subsequent vs. First)** | First session | 0.50 | 0.32 | 0.68 | <.001 |
| **Cumulative Stress (1.5 SD)** | - | -0.01 | -0.08 | 0.06 | .79 |
| **Session × Within-Person Stress** | - | 0.0002 | -0.0001 | 0.001 | .21 |

Random Effect for Intercept, σ = 0.77; Random slope, σ = 0.12. Note: Unstandardized effects (Est.) reported.

Table S10. Linear Mixed Effects Model Results for Episodic Memory

| **Variable** | **Reference Group** | **Est.** | **Lower**  **95% CI** | **Upper**  **95% CI** | **p-value** |
| --- | --- | --- | --- | --- | --- |
| **Between-Person Stress** | - | -0.0001 | -0.001 | 0.001 | .82 |
| **Session Number** | - | -0.0004 | -0.002 | 0.001 | .59 |
| **Within-Person Stress** | - | 0.0001 | -0.001 | 0.001 | .89 |
| **Education (centered)** | - | -0.001 | -0.01 | 0.01 | .71 |
| **Sex (female vs. male)** | Male | 0.03 | 0.001 | 0.07 | .05 |
| **Age (centered)** | - | -0.01 | -0.01 | -0.003 | < .001 |
| **Time of day (evening vs. afternoon)** | Afternoon | -0.01 | -0.023 | 0.003 | .14 |
| **Time of day (morning vs. afternoon)** | Afternoon | 0.01 | -0.01 | 0.02 | .33 |
| **Session (Subsequent vs. First)** | First session | -0.06 | -0.08 | -0.03 | < .001 |
| **Cumulative Stress (0.5 SD)** | - | -0.002 | -0.01 | 0.004 | .46 |
| **Session × Within-Person Stress** | - | -0.00002 | -0.0001 | 0.00003 | .37 |

Random Effect for Intercept, σ = 0.79; Random slope, σ = 0.007. Note: Unstandardized effects (Est.) reported.

Table S11. Linear Mixed Effects Model Results for Episodic Memory at 1 SD

| **Variable** | **Reference Group** | **Est.** | **Lower**  **95% CI** | **Upper**  **95% CI** | **p-value** |
| --- | --- | --- | --- | --- | --- |
| **Between-Person Stress** | - | -0.0002 | -0.001 | 0.001 | .71 |
| **Session Number** | - | -0.001 | -0.002 | 0.001 | .28 |
| **Within-Person Stress** | - | 0.0001 | -0.001 | 0.001 | .90 |
| **Education (centered)** | - | -0.001 | -0.01 | 0.01 | .68 |
| **Sex (female vs. male)** | Male | 0.03 | 0.002 | 0.07 | .04 |
| **Age (centered)** | - | -0.01 | -0.01 | -0.003 | < .001 |
| **Time of day (evening vs. afternoon)** | Afternoon | -0.01 | -0.02 | 0.003 | .14 |
| **Time of day (morning vs. afternoon)** | Afternoon | 0.01 | -0.01 | 0.02 | .34 |
| **Session (Subsequent vs. First)** | First session | -0.06 | -0.09 | -0.03 | < .001 |
| **Cumulative Stress (1.0 SD)** | - | -0.001 | -0.01 | 0.01 | .73 |
| **Session × Within-Person Stress** | - | -0.00003 | -0.0001 | 0.00003 | .35 |

Random Effect for Intercept, σ = 0.080; Random slope, σ = 0.0128. Note: Unstandardized effects (Est.) reported.

Table S12. Linear Mixed Effects Model Results for Episodic Memory at 1.5 SD

| **Variable** | **Reference Group** | **Est.** | **Lower**  **95% CI** | **Upper**  **95% CI** | **p-value** |
| --- | --- | --- | --- | --- | --- |
| **Between-Person Stress** | - | -0.0001 | -0.001 | 0.001 | .85 |
| **Session Number** | - | -0.001 | -0.002 | -0.0001 | .04 |
| **Within-Person Stress** | - | 0.0001 | -0.001 | 0.001 | .89 |
| **Education (centered)** | - | -0.001 | -0.01 | 0.01 | .67 |
| **Sex (female vs. male)** | Male | 0.03 | 0.0003 | 0.06 | .05 |
| **Age (centered)** | - | -0.01 | -0.01 | -0.003 | < .001 |
| **Time of day (evening vs. afternoon)** | Afternoon | -0.01 | -0.02 | 0.003 | .14 |
| **Time of day (morning vs. afternoon)** | Afternoon | 0.01 | -0.01 | 0.02 | .32 |
| **Session (Subsequent vs. First)** | First session | -0.06 | -0.09 | -0.03 | < .001 |
| **Cumulative Stress (1.5 SD)** | - | 0.004 | -0.01 | 0.01 | .40 |
| **Session × Within-Person Stress** | - | -0.00003 | -0.0001 | 0.00003 | .32 |

Random Effect for Intercept, σ = 0.081; Random slope, σ = 0.012. Note: Unstandardized effects (Est.) reported.

Figure S1. Mobile assessment interface for M2C2 tasks.

*Note.* Participant’s complete approximately 1-minute of survey questions, at the start of each study session, including questions about stress. Prices task is completed in two phases. Learning trials (phase 1) are followed by immediate delay forced choice recognition trials (phase 2).

Figure S2. Interaction Between Time in Study and Processing Speed Accuracy.

The figure displays predicted accuracy (y-axis) across session number (x-axis).

Dark purple = 0.5 SD above the mean; medium purple = at the mean; light purple = 0.5 SD below the mean. Predictions are based on the linear mixed model, with all other covariates held at their average or reference values. Lower within-person stress was associated with higher accuracy at the beginning of the study, but accuracy declined slightly over time. In contrast, higher within-person stress was associated with lower initial accuracy, which improved somewhat over sessions, suggesting a crossover interaction between stress and session number.
